# Subarachnoid hemorrhage as the initial imaging finding of atrial-esophageal Fistula: a case report highlighting diagnostic challenges

**DOI:** 10.3389/fcvm.2025.1594763

**Published:** 2025-07-18

**Authors:** Siwei Chen, Wei Sun, Yongan Sun, Lanqiu Yao, Qing Peng

**Affiliations:** ^1^ Department of Neurology, Peking University First Hospital, Beijing, China; ^2^ Department of Population Health, School of Medicine, New York University, New York, NY, United States

**Keywords:** atrial-esophageal fistula, subarachnoid hemorrhage, catheter ablation complications, delayed diagnosis, cerebral air embolism

## Abstract

Atrial-esophageal fistula (AEF) is a rare but life-threatening complication after catheter ablation. Neurological deficits represent the second most common clinical manifestation associated with AEF; however, diagnosis is often delayed because initial symptoms can be atypical and easily overlooked. Here we reported a case involving a 51-year-old male who presented with fever and headache three weeks after catheter ablation for atrial fibrillation. Initial cranial computed tomography (CT) showed right frontal subarachnoid hemorrhage (SAH) without aneurysm. As the patient's condition deteriorates, repeated imaging demonstrates worsening SAH, cerebral air emboli, and air signals in the left atrium. This case highlights the importance of considering AEF in patients with neurological deficits and recent cardiac ablation, even when initial imaging findings are atypical.

## Background

Atrial-esophageal fistula (AEF) is a rare condition associated with an extremely high mortality rate. The second most common clinical manifestation associated with AEF is neurological deficits (1). The primary neuroimaging findings include ischemic lesions and cerebral air embolism (2, 3). Delayed diagnosis often occurs because initial symptoms and signs are atypical and easily overlooked. However, to the best of our knowledge, cranial computed tomography (CT) findings showing subarachnoid hemorrhage (SAH) as the initial neurological manifestation of AEF have not previously been reported.

## Case presentation

**Figure 1:**
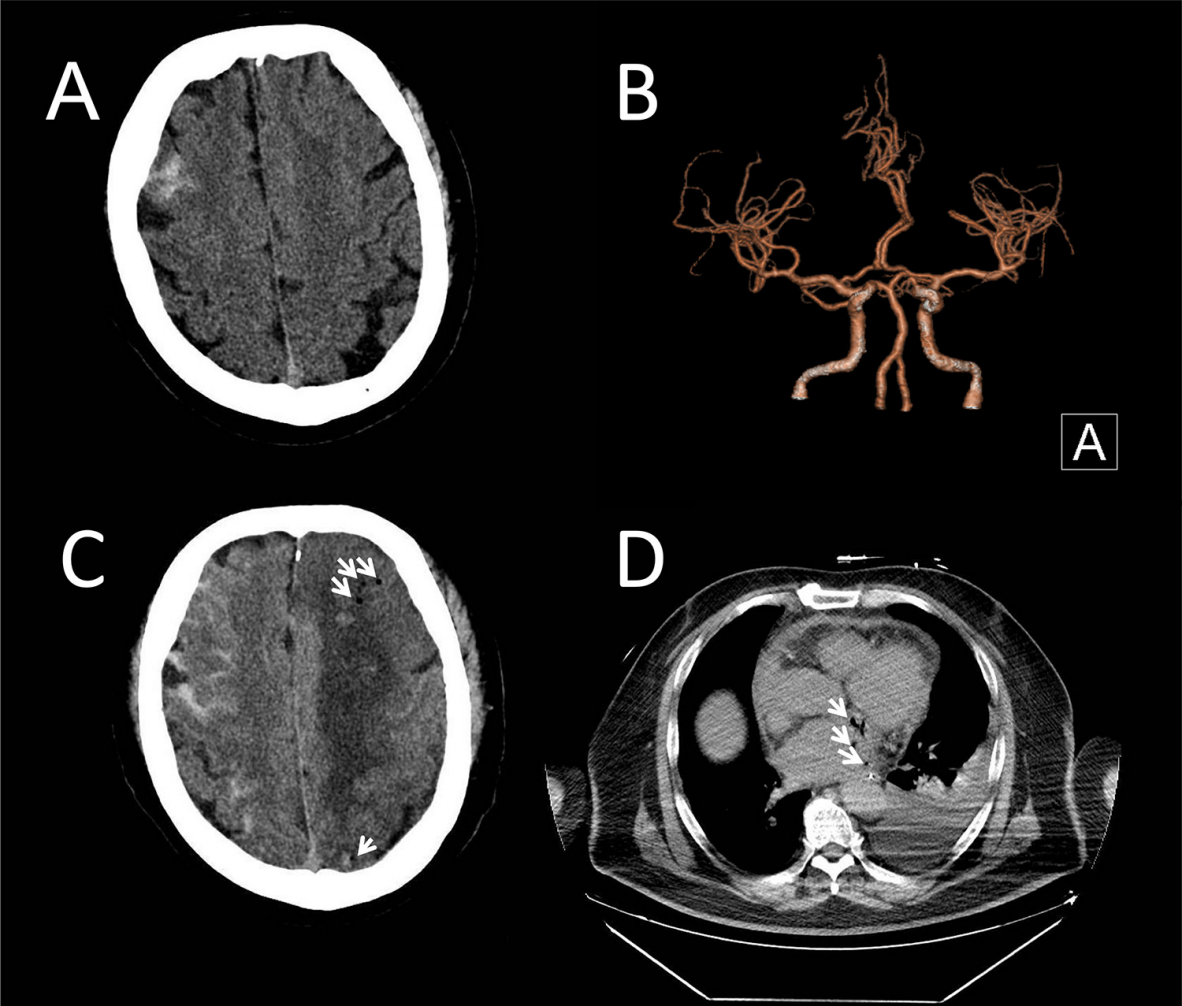
Brain CT and chest CT of the patient. **(A)** Initial cranial computed tomography (CT) revealed a right frontal subarachnoid hemorrhage. **(B)** CT angiography ruled out aneurysms. **(C)** Repeated cranial CT showed increased SAH, with air signals (arrows), infarcts and hemorrhagic densities in the left hemisphere. **(D)** Chest CT showed air signals (arrows) within and around the left atrium.

**Figure 2:**
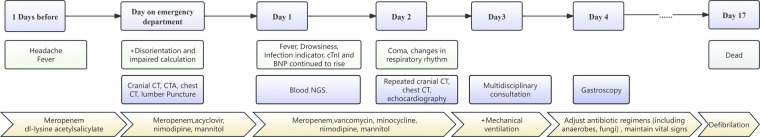
Timeline for development of this patient.

A 51-year-old male presented to our emergency department with fever and headache, three weeks after undergoing catheter ablation for atrial fibrillation. He had hypertension and diabetes for many years and took fosinopril and felodipine to lower blood pressure and acarbose to lower blood sugar. Atrial fibrillation was found 4 months ago. Rivaroxaban was taken for anticoagulation. Neurological examination on admission revealed disorientation and impaired calculation. Cranial CT and CT angiography showed right frontal SAH (Figure 1A) without evidence of an aneurysm (Figure 1B). Digital subtraction angiography (DSA) was not performed due to the small amount of early bleeding and the absence of evident aneurysm on initial CT angiography, as well as the patient's rapid clinical deterioration later. Chest CT revealed left pleural effusion and pericardial effusion. A lumbar puncture performed in the emergency department on the same day showed an opening pressure of 225 mmH_2_O. Cerebrospinal fluid (CSF) analysis revealed a nucleated cell count of 103 mm^3^ (13% mononuclear cells, 87% polymorphonuclear cells), a protein level of 0.45 g/L, glucose of 4.85 mmol/L, and chloride of 125.4 mmol/L. No bacteria, fungi, acid-fast bacilli, or cryptococci were detected on smear. For the central nervous system infection with unidentified pathogens, meropenem and acyclovir were administered. For the subarachnoid hemorrhage and intracranial hypertension, nimodipine and mannitol were initiated, along with blood pressure control. Anticoagulants were discontinued due to suspected infective endocarditis and the presence of intracranial hemorrhage. The next day, the patient remained febrile with headache, and inflammatory markers and cardiac enzymes showed progressive elevation. Neurological examination indicated that the patient was drowsy. CSF analysis via Next-generation sequencing (NGS) identified elevated levels of *Prevotella nanceiensis*. Minocycline and vancomycin were added to the antibiotic regimen, and acyclovir was discontinued. However, the next following day, the patient was in a mild coma. Repeated cranial CT showed increased SAH, with air signals and infarcts (Figure 1C). Repeated chest CT showed air signals in the left atrium and around (Figure 1D). Blood NGS confirmed bacteremia, identifying common Gram-positive and Gram-negative bacteria typically found in the oral cavity. Echocardiography indicated the presence of a neoplasm in the left atrium, with significant oscillation during the cardiac cycle, and partial movement into the left ventricle during diastole via the mitral valve. Gastroscopy confirmed the presence of an AEF. Considering the high surgical risk and family's wishes, conservative treatment was chosen over surgery. The patient died 17 days after admission due to sepsis and infective endocarditis. The detailed development of the disease can be seen in Figure 2.

## Discussion

Although AFE is rare, its consequences are severe, underscoring the importance of early diagnosis and intervention. Clinically, AEF typically presents within a few weeks after the ablation procedure, with symptoms such as fever, chest pain, and neurological deficits. The neurological manifestations of AEF include embolic stroke, seizures, transient ischemic attacks, coma, or mental abnormalities (4). Imaging studies typically show multiple embolic strokes or air embolism (4). The diagnosis of AEF is challenging due to its rarity and the nonspecific nature of its symptoms. Contrast-enhanced CT scans are commonly used for diagnosis, while repeated chest and head CT or magnetic resonance imaging (MRI) plays a crucial role in detecting abnormal findings. However, a high level of clinical suspicion remains necessary to avoid delays in treatment (1, 2). Therefore, in patients presenting with neurological deficits and a history of catheter ablation within 60 days (2) before admission, clinicians should remain vigilant for AEF even if initial imaging findings or symptom appear atypical.

In this case, we speculated that a microaneurysm had formed due to bacteremia, leading to SAH and initially presenting with fever and headache. *Prevotella nanceiensis* is one of the core anaerobic species in the oral microbiome (5). We inferred that bacteremia from oral flora (e.g., *Prevotella spp.*) may promote infectious aneurysm formation via endothelial inflammation, potentially explaining SAH in AEF-related sepsis (6). Both blood and CSF NGS results further supported the presence of bacteremia and the potential formation and rupture of an aneurysm. Clinicians initially considered infective endocarditis and initiated active treatment but did not recognize that AEF had already developed. As the patient's condition deteriorated, repeated brain and chest CT scans revealed intracranial air, ultimately identifying the true etiology. The sequential occurrence of subarachnoid hemorrhage (SAH) and air embolism reflects an evolving pathophysiology: Sepsis initially triggered the formation and rupture of an infectious intracranial microaneurysm, causing SAH. The infection-induced vascular injury disrupted the blood-brain barrier. Subsequent enlargement of fistulous tracts permitted progressive air entry. As demonstrated in Figure 1C, bilateral hemispheric involvement was observed: the right hemisphere showed predominant SAH, while the left exhibited more pronounced infarction, parenchymal hemorrhage, and air densities. The involvement of non-contiguous vascular territories across both hemispheres further supports the possibility of cardiogenic embolism.

The clinical treatment of infectious intracranial aneurysm (IIA) is a complex and challenging process involving multidisciplinary collaboration. IIA is a rare but serious vascular lesion that is often associated with infective endocarditis. Treatment strategies typically include antibiotic therapy, neurosurgery, and endovascular therapy. Antibiotic therapy is the cornerstone of treatment for all patients with IIA. Studies have shown that appropriate antibiotic therapy can lead to the reduction or disappearance of aneurysms in certain cases (7, 8). However, the effectiveness of antibiotic therapy may be limited by the size and shape of the aneurysm; larger aneurysms or those with a cystic appearance may not respond well to antibiotic therapy (7). Besides, neurosurgical procedures and endovascular treatments are crucial for managing IIA, whether the aneurysm has ruptured or not ruptured but is unresponsive to antibiotic therapy (8). Since no aneurysm manifestations were found in the CTA of this patient, antibiotic treatment was the main approach.

The management of AEF often requires a multidisciplinary approach, involving both cardiac and thoracic surgeons. Surgical intervention is generally considered the most effective treatment, with procedures such as patch repair of the left atrium and primary repair of the esophagus being common. The use of cardiopulmonary bypass during surgery may improve outcomes by facilitating more debridement and repair of the affected areas (9). Despite surgical intervention, the prognosis remains poor, with mortality rates reported to be as high as 71% in some studies (4).

Preventive strategies during catheter ablation, such as esophageal temperature monitoring and proton pump inhibitors use, have been explored to reduce the risk of AEF; however, their effectiveness remains uncertain (10). More recently, novel approaches such as esophageal cooling during radiofrequency delivery and pulsed field ablation (PFA)—a non-thermal energy source—have demonstrated significant promise in minimizing esophageal injury and AEF risk (11, 12). The advent of PFA, in particular, may fundamentally alter the safety profile of atrial fibrillation ablation by eliminating thermal collateral damage to adjacent structures (12). These technological advances hold potential to substantially reduce, or even eradicate, such devastating complications in the near future.

The rarity of AEF, along with its variable presentation and outcomes, make it challenging to establish standardized guidelines for prevention and management (13). Unfortunately, the patient's condition deteriorated rapidly, presenting with severe infection and unstable vital signs. Following multidisciplinary consultation, surgical intervention was deemed unfeasible. After extensive discussions with the family, continued medical management was pursued; however, the prognosis remained poor. This outcome further highlights the lethality of AEF.

This is the first reported case of isolated subarachnoid hemorrhage (SAH) as the initial presentation of AEF to our knowledge. However, the lack of surgical/pathological confirmation of the fistula and retrospective diagnosis constitute limitations. Although gastroscopy and serial radiological evolution were highly suggestive of AEF, future cases require direct histopathological correlation. Moreover, the proposed mechanisms underlying SAH occurrence remain speculative without histopathological verification, which further limits this report.

## Conclusion

SAH may present as the initial imaging finding in AEF. Clinicians must remain vigilant for AEF in patients with neurological deficits and a recent history of cardiac ablation, even if initial imaging is atypical. Repeated imaging and multidisciplinary collaboration are critical for timely diagnosis.

## Data Availability

The original contributions presented in the study are included in the article/Supplementary Material, further inquiries can be directed to the corresponding author.
